# The Regulatory Network of *Pseudomonas aeruginosa*

**DOI:** 10.1186/2042-5783-1-3

**Published:** 2011-06-14

**Authors:** Edgardo Galán-Vásquez, Beatriz Luna, Agustino Martínez-Antonio

**Affiliations:** 1 Departamento de Ingeniería Genética, Cinvestav, Km. 9.6 Libramiento Norte Carr. Irapuato-León 36821 Irapuato Gto. México

## Abstract

**Background:**

*Pseudomonas aeruginosa *is an important bacterial model due to its metabolic and pathogenic abilities, which allow it to interact and colonize a wide range of hosts, including plants and animals. In this work we compile and analyze the structure and organization of an experimentally supported regulatory network in this bacterium.

**Results:**

The regulatory network consists of 690 genes and 1020 regulatory interactions between their products (12% of total genes: 54% sigma and 16% of transcription factors). This complex interplay makes the third largest regulatory network of those reported in bacteria. The entire network is enriched for activating interactions and, peculiarly, self-activation seems to occur more prominent for transcription factors (TFs), which contrasts with other biological networks where self-repression is dominant. The network contains a giant component of 650 genes organized into 11 hierarchies, encompassing important biological processes, such as, biofilms formation, production of exopolysaccharide alginate and several virulence factors, and of the so-called quorum sensing regulons.

**Conclusions:**

The study of gene regulation in *P. aeruginosa *is biased towards pathogenesis and virulence processes, all of which are interconnected. The network shows power-law distribution -input degree -, and we identified the top ten global regulators, six two-element cycles, the longest paths have ten steps, six biological modules and the main motifs containing three and four elements. We think this work can provide insights for the design of further studies to cover the many gaps in knowledge of this important bacterial model, and for the design of systems strategies to combat this bacterium.

## Background

*Pseudomonas aeruginosa *is a metabolically versatile Gram-negative bacterium, able to express a wide variety of virulence factors. These allow *P. aeruginosa *to grow in soil and marine habitats, as well as on plant and animal tissues. It is also a significant source of bacteraemia in burn victims, urinary-tract infections, hospital-acquired pneumonia and predominant cause of morbidity and mortality in cystic fibrosis patients [1]. All these makes *P. aeruginosa *the most studied bacterial model regarding the control of pathogenic determinants and the third bacterial model more studied with respect to their molecular biology -after *Escherichia coli *and *Bacillus subtilis*-. The genome sequence of *P. aeruginosa *strain PAO1 was reported in 2000 [1], and since then numerous databases and genomic resources have been implemented to study their molecular and pathogenic biology [2-4].

It is well know the importance of gene regulation on the organisms' performance as this process defines their metabolic, adaptive and pathogenic capabilities. In this work, we report a collection of known regulatory network interactions connecting transcription factors (TFs), sigma factors (σ), and anti-sigma factors to their target genes in *P. aeruginosa*. This transcriptional regulatory network (TRN) constitutes the third largest one of any bacteria reported to date. We proceed to analyze the main topological properties of this network and the main functional interactions among their regulatory components. We hope these results will provide insights and guide future studies to increase our knowledge on this important bacterium.

## Results and discussion

### The transcriptional regulatory network (TRN) of *Pseudomonas aeruginosa*

**Figure 1:**
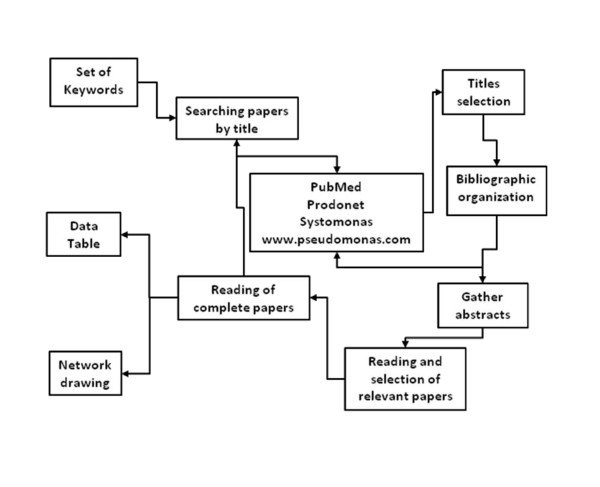
**Strategy to compile the network of *P. Aeruginosa***. General strategy for gathering information about transcriptional regulation used to construct the TRN of ***P. aeruginosa***

**Figure 2:**
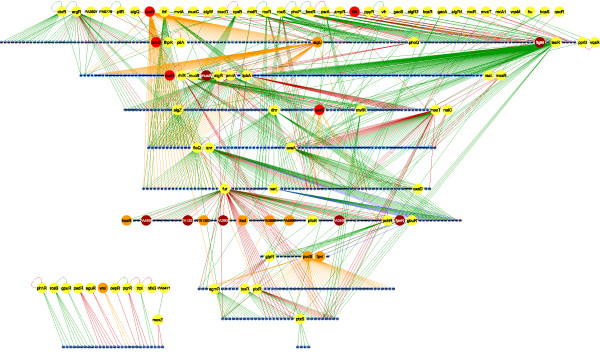
**The TRN of *P. Aeruginosa***. Different types of genes are represented by nodes with different colors; TF (yellow), σ (red), ECF (orange) anti-σ factors (brown), and non-regulatory genes (blue). Arrows represent the modes of regulation; transcriptional activation (green), transcriptional repression (red), transcriptional dual regulation (blue), and undefined (gray); transcription by σ (orange) and σ control by anti-σ (black). The network was drawn using the Cytoscape software [28]. For a better clarity, a poster version of this figure is available as additional file 2.

With the aim of summarizing all the documented action of the regulatory machinery over the genes encoded in the genome of *P. aeruginosa*, the available published data was searched using a combined strategy: 1) regulatory interactions from dedicated biological databases were extracted [2-4] and, 2) searches in the original literature were performed (see Figure 1 for the general strategy). Both sources of information were verified by analyzing the corresponding papers. Methods frequently used to study transcriptional regulation included microarray analyses and their validation, promoter activity through transcriptional fusions, RT-PCR, EMSA assays and DNA-foot printing [Additional file 1 which contains a complete description of the network interactions along with their experimental evidences and references]. As of May 2010 our curated network consisted of 1020 regulatory interactions among 690 genes products, including 76 transcription factors, 14 sigma factors (nine of these defined with extra-cytoplasmic functions -ECF-), 7 anti-sigma factors, and 593 target genes (Figure 2, a poster version of this figure is available as additional file 2). Given the 5,570 predicted protein coding genes of *P. aeruginosa *PAO1 (the strain on which most of the network reconstruction is based) our network represents roughly 12% of these genes. On the other hand, the regulatory machinery predicted in this bacterium comprises around 500 proteins: 26 sigma factors (one σ^54^, eight σ^70^, and 17 of ECF families), the rest corresponding to transcription factors distributed in at least 44 families [5]. This network then, represents roughly 54% of sigma factors and 16% of all the TFs encoded by this bacterium. In the following sections we will report the structural and the functional properties of this network.

### Topological description of the TRN in *P. aeruginosa*

#### Degree distribution

**Figure 3:**
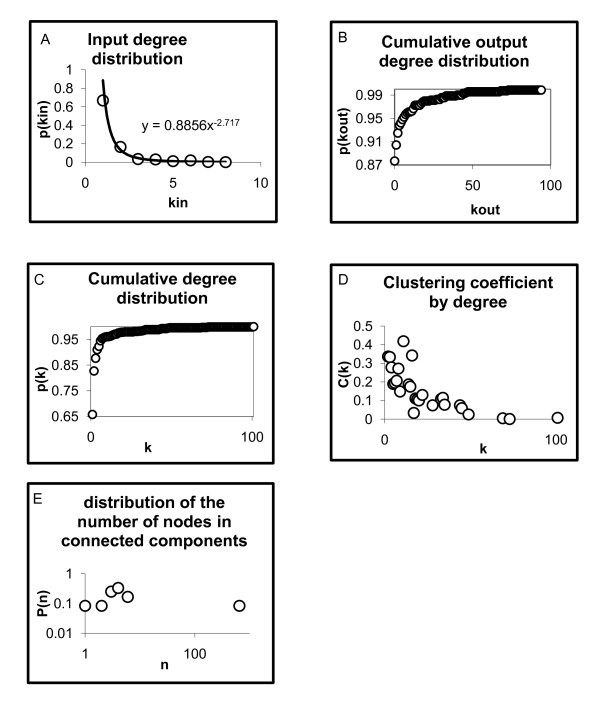
**Topological measurements of the TRN of *P. Aeruginosa***. Topological measurements of the TRN of ***P. aeruginosa ***considering the entire network with 690 genes and 1020 regulatory interactions; A) input degree distribution, B) cumulative output degree distribution in the undirected graph, C) cumulative degree distribution, D) clustering coefficient by degree, and E) the distribution of the number of connected components in the network, see details in the text and Methods section.

In network and graph theory, the degree (*k*) of a node (gene) is defined as the number of interactions that it has with other nodes. Here we determined the gene's mean degree as the arithmetic average of all the node-degrees (*k*) in each network [Additional file 1]. This result implies that each gene in the TRN of *P. aeruginosa *is connected, on average, with 3 other genes. In directed networks, as in the case of regulatory networks, we can define input (*kin*) and output degree (*kout*) as the number of arrows that enter and leave genes respectively, which corresponds to the number of TFs that regulate a certain gene, and the number of genes that a TF is regulating. The degree distribution gives the probability *P(k)*, of finding a node with degree *k *[6]. This measure quantifies the diversity of gene-degrees in a network and allows determining which theoretical network is more similar to the type of network we are working with (i.e. classical random networks, scale free, small-world, etc. [7,8]). Some authors claim that biological networks present a well-known distribution closer to a power law  which indicates that a few genes are highly connected (they are called hubs), while most of them have low connectivity [9]. The constant *A *ensures that the *P(k) *values are normalized towards 1, and γ is a parameter that provides information about the network structure; for networks with γ>3 a lot of the properties for scale-free networks are not present, which are present for 2<γ<3, where there is a hierarchy in the degree of nodes, from the most to less connected ones. However, for γ= 2, the highest degree node influences a large fraction of all nodes [9]. In the case of the TRN of *P. aeruginosa*, we find A = 0.8856 and, 2<γ<3 for the input degree distribution (Figure 3A), but without a good trend of this type for the output and overall degree distributions. Because of this, we show instead their corresponding cumulative distributions *P(kout ≤ Kout), P(k ≤K)*, (Figures 3B and 3C respectively). Overalls the fact that a few genes are highly connected remains valid.

#### Clustering coefficient

The clustering coefficient *C*, is a measure that indicates the probability that two genes with a common neighbor in a graph are also interconnected; that is to say, the clustering coefficient quantifies what so much the local neighborhood of a gene is as member of a group of genes. It is common for networks to exhibit a decreasing value of *C(k) *with respect to the degree *k*, such that in small groups or modules of genes the elements are well connected, but as the group increases in size the elements are progressively less connected. The regulatory network of *P. aeruginosa *shares this general clustering property in (Figure 3D).

#### Connectivity

Connectivity in a network refers to the associations between every pair of genes.

Connections can be via a direct link or indirectly through a series of intermediate interactions. Connected components are defined for undirected networks, and give us information about how much are connected the elements in a network and their modular structure. Sometimes it is necessary to consider the network as undirected, since it allows us to capture different types of information to perform a better analysis. In the case of the TRN in *P. aeruginosa *there are 12 connected components, with one giant component containing 650 genes, while the rest contain at most six genes. Each connected component in the TRN possesses at least one TF or σ. A skeleton of 65 TFs and 13 σ maintain cohesive this giant component (the 12% of its components). We consider that a connected component is composed by *n *nodes, and calculate the relative frequency *P(n) *for every possible *n*, which give us the distribution of the number of nodes in a connected component (Figure 3E), [see also Additional file 1].

### Functional organization of the regulatory network

In order to discern the functional organization of a regulatory network we can study the following aspects of the TRN: i) the regulatory mode and connectivity of each component of the transcriptional machinery and, ii) the manner in which endogenous and exogenous information, relevant for transcriptional regulation, enter and pass through the regulatory machinery until conclude on promoters of target genes. All these computes should be associated with the biological functions of the respective genes. In this sense, some interesting findings in the TRN of *P. aeruginosa *are discussed below.

#### Activation is the dominating activity in the TRN

**Figure 4:**
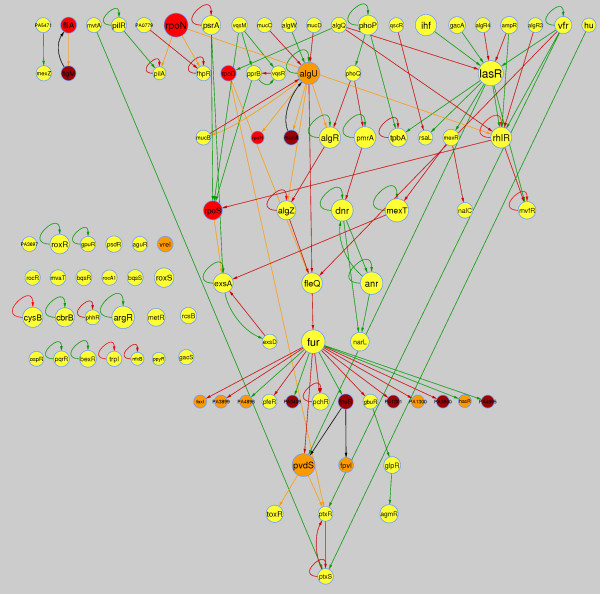
**Transcriptional machinery sub-network in *P. aeruginosa *(TF and sigma factors)**. Nodes and arrows are the same as in Figure 2 with the exception that it lacks of the non-regulatory genes. The network is presented in a hierarchical structure, in order to better appreciate the hierarchical organization among TFs and σ.

Analysis of the mode of regulation (excluding interactions by σ and anti-σ) showed, that activation is by far the dominating regulatory activity in *P. aeruginosa *(Table 1). For comparison, the *E. coli *network also shows a higher tendency for activation instead of repression although in *P. aeruginosa *this difference is more pronounced. This dominant mode of regulation is also evident in the sub-network consisting of TFs and σ factors in *P. aeruginosa *(Figure 4), and the same was also observed in *E. coli *[10]. Positive regulation in these networks might explain why once a biological process is triggered it can run from the beginning to the end of defined regulatory pathways, perhaps giving place to conditioned memory as has been observed in *E. coli *and *Saccharomyces cerevisae *[11,12].

**Table 1:** Network statistics

	Whole network	Sub network
Number of TFs	76	76
Auto-regulations	29 (38.15%)	29
Positive auto-regulations	16 (55.17%)	16
Negative auto-regulations	13 (44.82%)	13
Regulatory arrows	1020	122
Positive arrows	779 (76.37%)	70 (57.37%)
Negative arrows	218 (21.35%)	52 (42.62%)
Dual arrows	11 (1.07%)	0
Unknown arrows	12 (1.17%)	0
Average path length	4.08	2.13
Maximum out degree	95 (lasR)	14 (fur)
Maximum in degree	8 (rhlI)	7 (rhlR)

The number and sign of interactions and other topological measures for the whole network and the sub-network, which includes only the regulatory machinery of*P. aeruginosa*, are shown (Figure 4).

#### Most of the TFs are positively auto-regulated

The mode in which regulatory genes are auto-regulated is important for network dynamics. It is known that auto-repressions are important controllers to keep homeostatic levels of biological functions, while auto-activation is a condition to reach multiple steady states and differentiation [13]. Operatively, the TFs are important points of genetic control since they are the master switches whose regulatory activity extends over many genes. Consequently, it is not surprising to realize that nature has developed self-regulation on these genes as a quick and effective mode of control over a wide range of physiological processes. In contrast to what happens in *E. coli *and *B. subtilis*, auto-regulation is mostly positive for the TFs of *P. aeruginosa *(Table 2 and Figure 4). Given that negative self-regulation maintains homeostasis, is normal to observe this mode as dominant in regulatory networks as in *E. coli *[10,14], and *B. subtilis *(Galán-Vásquez *et al*, unpublished observations), with the exception of developmental processes for biofilm and flagella formation in *E. coli *where self-activation is enriched [10]. This observation is in agreement with the postulation that auto-activation is normally found at the core of differentiation and development processes. Dynamic analysis shows that auto-activation causes a slow and delayed response compared to auto-repression and simple regulation [15]. It is supposed that this delayed response gives time enough for regulation pass through different check points, before the differentiation processes goes ahead. Given the bias of knowledge toward the study of only a minor part of the network in *P. aeruginosa *(virulence and pathogenic processes), it is premature to conclude whether that distinction should be a property of the whole network, or if, it mainly represents an evolutionary design for the execution of pathogenesis and virulence actions by this bacterium. Using the database of Reciprocal Best Hits (RBH) orthologs genes in bacteria [16], we found orthologs for seven regulatory genes from *P. aeruginosa *in *E. coli*, in both organisms with experimental evidence about their mode of self-regulation [Additional file 1]. From these, the three negatively auto-regulated genes in *P. aeruginosa *conserve this mode of self-regulation in *E. coli*. For the remaining four positively auto-regulated in *P. aeruginosa*, one conserved their positive mode of auto-regulation in *E. coli *and the rest three changes; two are dual and one self-regulate negatively in *E. coli*. Although this information is scarce, it might be possible that positive auto-regulation in TFs might be effectively selected in *P. aeruginosa*.

**Table 2:** Top ten most influencing regulators in the TRN of *P. aeruginosa*

Transcription factor	TFs and σ regulated (excluding self-regulation) *TFR*	Total of genes regulated *GR*	Type of σ used by the regulated promoters *SF*	Number of TFs used as co-regulators *CR*	*G *coefficient
lasR	6	89	3	20	0.155527032
Fur	14	54	3	6	0.114938218
mexT	1	46	3	15	0.10771137
Vfr	4	3	4	12	0.09930047
algR	1	29	3	14	0.097211089
Anr	2	42	2	12	0.086724434
Ihf	1	29	2	14	0.085306327
ptxR	1	11	3	12	0.082955889
rhlR	2	25	2	13	0.082890819
algW	1	1	3	7	0.062073371

The highest values of G coefficient obtained from the TFs of whole network are shown.

#### Path lengths

**Figure 5:**
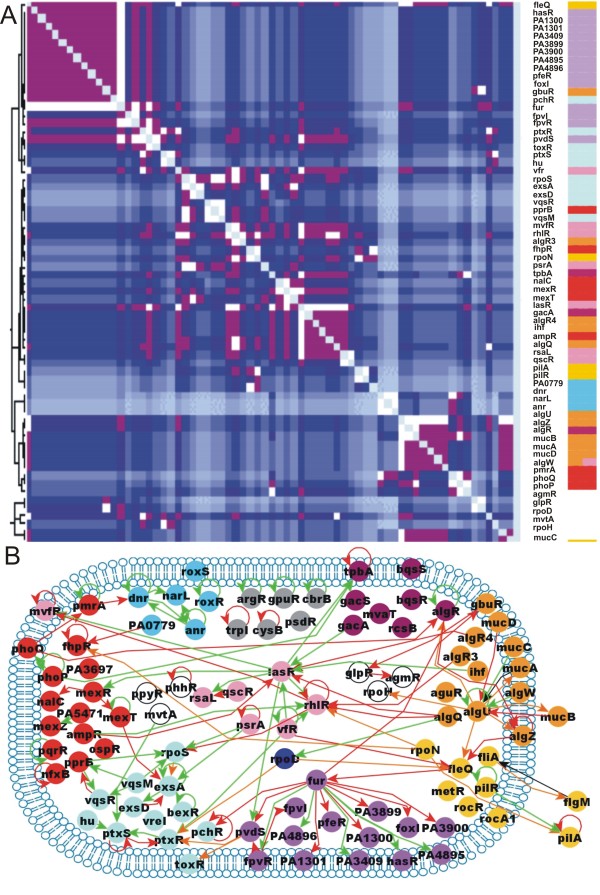
**Functional modules**. Determination of biological modules more represented in the regulatory machinery of ***P. aeruginosa***. A) Using a short path metric distance 1/***D^2 ^***where ***D ***is the distance between two nodes. White color represents interactions with ***D = 1***, purple color shows the interactions with ***D = 2***, interactions with ***D = 3 ***are displayed in blue, with diminishing intensity as distance increases until a light blue color, representing interactions that do not exist. B) The major clusters of biological functions were also identified by analyzing the scientific literature: quorum sensing (pink), alginate biosynthesis (orange), iron metabolic (violet), nitrogen metabolic (cyan), motility (yellow), antibiotics resistance (red), expression of virulence factors (sky blue), biofilm formation (purple) and amino acid metabolism (gray). For the sake of comparison between both approaches, the same colors were used for the regulatory machinery in both figures.

A path in a TRN refers to a chain of regulatory interactions between the genes constituting it. The longest path in the TRN of *P. aeruginosa *consists of 11 steps. This is almost the same size than in their *E. coli *counterpart where the longest paths include 14 steps. The size of paths in *P. aeruginosa *is interesting if we consider that the TRN is far from complete, therefore it is reasonable to expect that longer paths can be found as the network will be further characterized. The longer regulatory path in *P. aeruginosa *goes along biological processes that include alginate biosynthesis, iron metabolism and pioverdine synthesis, implying that these processes could be physiologically connected (Figures 2 and 5). The most frequent path-size (1102 times) found in the *P. aeruginosa *TRN consists of five steps [Additional file 1].

Short paths are common in metabolic and signal transduction networks, since this arrangement ensures fast and efficient response to changes in food use and to environmental perturbations [6]. The longest paths in the TRN of *E. coli *include regulatory process for biofilm formation and flagella assembly [10], both of these are considered development processes and are also amongst the longest paths in *P. aeruginosa*.

#### Cycles

**Figure 6:**
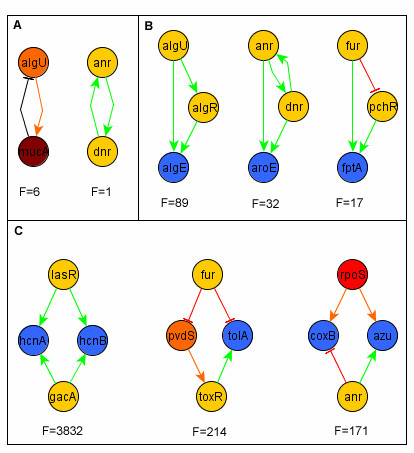
**Cycles and network motifs in the TRN of *P. aeruginosa***. A) The most abundant two-node motif are the negative feedback with a frequency (F) of six in the network. The three most abundant motifs of three and four nodes are shown, B) the most abundant motif of three nodes is the coherent feed forward loop with a frequency of 89, C) the most abundant four-node motif is the bi-fan with a frequency of 3832. The frequency of each cycle and motif was compared with the average of those found in 1000 random networks.

Biological systems frequently contain positive or negative feedback loops. Multi-element biological cycles (with two or more components) can also be positive or negative (depending on the product of the signs of the constitutive interactions).The existence of positive cycles is a necessary condition to have multiple steady states or attractors. Negative cycles are important to keep homeostasis, since they maintain the system functioning through periodic orbits. Besides self-regulation, multi-element circuits in a TRN can be defined as self-enclosed paths. Until now, excluding self-regulation, there are only seven cycles with two regulatory factors in *P. aeruginosa *(Figure 6A); six negatives (*alg*U-*muc *A and *alg*U-*muc*B, for the control of alginate synthesis; *exs*A-*exs*D, for the control of secretion systems; *flg*M-*fli*A, for motility control; *ppr*B-*vqs *R which enhance exotoxin A production and; *ptx*R-*ptx *S that control protease and pyocyanine synthesis) and one positive (*anr-dnr*, for the control of aerobic/anaerobic respiration), and no cycles with more TF-elements are present in the TRN. The dynamics of positive and negative two-node cycles has been widely studied. They represent important core components for network dynamics acting as robust switches to respond to signals from environmental conditions [17].

#### Motifs

A motif in a TRN is a topological structure that is more frequented than expected [18]. The most represented motifs in the *P. aeruginosa *network are those formed by three and four genes (Figure 6), [Additional file 1]. Previous research suggests that motifs represent elements for optimal network design given their relationship with the network dynamics and structural stability. The prevalence of certain types of motifs has been considered a product of the evolution acting on the organization of biological networks [19-21]. In particular, motifs such as feed-forward loops (FFL; networks with three vertex, composed of two input transcription factors, one of which regulates the other, both jointly regulating a target gene) have a higher abundance in TRN than expected from random networks with the same number of nodes and arrows [18,22]. The dynamic behavior of feed-forward loops has been extensively analyzed [23]; these studies revealed that FFL have two main functions: a) to speed up the response time of the target gene (incoherent FFL, when the signs of the direct and indirect regulation are opposites) and, b) to act as sign-sensitive delays for one of the two TFs (coherent FFL, with the same sign for both the direct and indirect regulation). Considering all the biological process where they participate, FFL are also implicated in pulse generation and cooperativity. In *P. aeruginosa *the most common motifs are those of three nodes known as coherent feed-forward loops [23], where the sign of the interactions is the same, positive in this case (Figure 6B). This type of motif is present 89 times in the *P. aeruginosa *regulatory network. Additionally, we found that the most common motifs of four nodes, which occurred 3832 in the network, are those known as bi-fan, where two TFs each positively co-regulate to two target genes (Figure 6C). This motif is also frequent in other organisms such as *S. cerevisiae *and *E. coli *[24].

#### Hierarchical organization of the TRN of P. aeruginosa

A hierarchical organization is given by a directed informational flux, beginning from the most influencing regulators. In this way, the TFs constitute the skeleton and the non-regulatory genes are the leaves in a hierarchical network (Figure 2 andAdditional file 1). The first level is populated by 33 TFs and 2 sigma factors. The origons [25], which are the points of informational inputs into the network, are set at this level. The second level is the most populated level but includes a high proportion of non-regulatory genes. Most of σ are set at higher levels, except for those involved in iron metabolism, which, as also observed for *E. coli*, are in the lowest levels as dedicated sigma factors for specific functions.

#### Co-regulation

Promoter and regulatory regions are zones in transcription units where regulatory information is integrated. This regulatory integration is evidenced by the presence of DNA-binding sites for multiple regulators or promoters for different σ respectively. In the TRN of *P. aeruginosa*, regulators of level one can co-regulate with regulators of any other level and this co-regulatory activity diminishes as TFs are set lower in the network hierarchy. Regulators that frequently co-regulate with other regulators (in parenthesis the number of co-regulations) are: *lasR(20*), *mexT(15), algR(14), ihf(14), rhlR(13), anr(12), ptxR(12)*. On the other hand, the most regulated genes are (in parenthesis the number of TFs regulating it): *rhl*I(8), *rhl*AR(7), *alg*D(7), *alg*44(6), *alg*8(6), *alg*A(6), *alg*E(6) *alg*F(6), *alg*G(6), *alg*I(6), *alg*J(6), *alg*K(6), *alg*L(6), *alg*X(6), *hcn*A(6), *las*R(6), *alg*U(6). It is interesting that the most regulated genes in both *E. coli *and *P. aeruginosa *encode for TFs, FhlCD (flagella synthesis) and RhlI (quorum sensing) respectively.

#### Most influencing regulators in the TRN of P. aeruginosa

The most influencing regulators in a regulatory network are called "global regulators" and are defined by a series of operative properties, including: i) they should regulate a large number of genes; ii) should regulate other sigmas and regulators; iii) should co-regulate together with many TFs and, iv) their target genes should have promoters using more than one kind of σ [26]. All these properties were computed for regulators found in *P. aeruginosa *(see Methods section) and the top ten are shown in Table 2. A coefficient *G *was introduced here, which indicates if a regulator is more or less global taking into account the regulatory criteria mentioned above. The most influencing regulators in *Pseudomonas *have a lower qualification than the corresponding seven global TFs in *E. coli*. This might be due to the limited knowledge of the transcriptional regulation in *P. aeruginosa *compared to *E. coli*.

#### Biological processes in P. aeruginosa TRN

Defining functional modules in a formal computational way is a difficult task. However, it has been shown that employing a simple metric of shorter distances among TFs in the *E. coli *network, it might be possible to recover modules with a good approximation to those that are manually defined, on the basis of the knowledge of biological functions of their products [27]. In this work we used this metric for the TFs and σ -anti-σ sub-network of *P. aeruginosa *(Figure 4), and get the following biological modules: alginate biosynthesis, quorum sensing, iron capture and metabolism, production of virulence factors, antibiotic resistance and motility (Figure 5A). This finding was corroborated by manual inspection of TFs participating in the same biological processes (Figure 5B). It is clear that the processes that are more thoroughly studied in *P. aeruginosa *correspond to those related to pathogenesis and virulence properties while little attention has been given to biological processes, such as, central metabolism, membrane biogenesis, cell-division, etc. Most of the best-studied biological processes are connected, beginning from alginate biosynthesis to quorum sensing, and from there to those involved in the production of virulence factors. Additionally, there is a directed regulatory connection from alginate biosynthesis to iron metabolism and to some mechanisms of antibiotic resistance (Figure 5B). Since these processes act cooperatively during infection and pathogenesis, it is very important to give a detailed characterization of *P. aeruginosa *regulatory network. The latter may lead to the development of strategies to disrupt its connectivity, thus, possibly decreasing the pathogenicity of this bacterium.

## Conclusions

Here we report the topological and functional organization of the third largest regulatory network in bacteria. From our analysis, it is evident that the study of regulation in *P. aeruginosa *is biased towards particular biological processes, involved in pathogenesis and virulence. These processes include alginate and biofilm formation, production of virulence factors and antibiotic resistance, many of which are coordinated by quorum sensing in the bacterial population. Current data suggests, that motility, iron metabolism and anaerobic respiration might be less connected to these processes by now. All these processes are connected in the network via a hierarchical organization with 11 levels, and the connected parts of the network form a giant component with 650 genes, the 10% of them corresponded to TFs. Overall, the network has degree distribution and structural organizations as other biological networks known to date. A peculiar property of this network is the fact that its TFs are mainly auto-activated. This is the first time this mode of self-regulation is reported as dominant in a bacterial TRN. It remains to be revealed whether this property is really a characteristic of the entire network in this bacterium, or is just is property of this part of the network, which clearly controls adaptive, pathogenic and virulence processes. As it can be observed, regulatory information related to several important biological processes of *P. aeruginosa *is lacking; for instance, the regulation on the uptake of carbon sources and their metabolism, amino acid biosynthesis or cell-division. This bias makes difficult a complete analysis on the regulatory network of this bacterium and better compare it with regulatory networks of bacteria most characterized such as *E. coli *or *B. subtilis*. It might be that studying basic biological functions on this organisms we can understand the basis of their versatile metabolism, adaptiveness and pathogenecity. In special it is lacking the knowledge of the activity of the housekeeping sigma and transcription factors controlling activities of central metabolism. Because of this it will be very important for the community working on the biology of *P. aeruginosa *to study additional biological processes in order to have a more complete picture of the regulatory network in this bacterium. We hope this analysis will give insights in this direction to guide future work, with the aim of covering the many gaps of knowledge on this important bacterial model.

## Methods

### Biological data and representation

The general strategy for the curation of regulatory interactions is shown in Figure 1. Briefly, we searched PubMed with relevant key words, such as: *P. aeruginosa*, sigma or transcription factor, transcriptional regulation, etc. Data on regulatory networks were obtained from the literature and compiled in an Excel table including experimental evidence and references. The Additional file 1 shows the complete information for the interactions of the entire network. The regulatory interactions were drawn in a form of network using the Cytoscape software [28].

### Transcriptional machinery sub-network

With the aim of analyzing the regulatory behavior of the transcriptional machinery of *P. aeruginosa*, the regulatory interactions present only among TFs, sigmas and anti-sigmas from the whole network were extracted (Figure 4).

### Computational analysis of the regulatory network

All the computational analyses on the network were made using the Octave free software http://www.octave.org. Analyses of degree, centrality, clustering coefficient, connectivity, cycles, paths and hierarchical levels were made according to previous definitions and following the approach as in [10]. Motif determination was made following the work by Uri Alon and coworkers calculating the probability of finding the same motif in a random network as the average of the motifs found in 1000 randomized networks, maintaining the same number of nodes, edges and the proportion of the type of regulatory interactions (positive, negative, dual) [24].

### The G coefficient for global regulators

We computed the coefficient *G*, which indicates the global activity of a TF in a TRN as follows:

Where, *N_TF _*indicates the total number of TFs (in the known network in each case), *N_G _*is the number of non-regulatory genes, and *N_SF _*is the number of sigma factors in the whole network. Additionally, *TFR *and *GR*, represents the number of TFs and non-regulatory genes regulated by each TF, respectively; *SF *represents the distinct sigma factors used by the promoters of genes regulated by each TF; and *CR *represents the number of TFs each TF co-regulates with.

### Determination of biological modules/processes in the regulatory machinery network

With the aim of determining biological modules in the TRN we used a shortest path metric criteria among TFs and sigmas (we used the relation 1*/D^2 ^*where *D *is the distance between two nodes), as reported for *E. coli *[27]. Additionally, we manually grouped TFs and sigma factors in agreement to the functional classification of their regulated genes [4].

## List of abbreviations

TF: transcription factor; σ: sigma factor; ECF: extra-cytoplasmic factors; TRN: transcriptional regulatory network.

## Competing interests

The authors declare that they have no competing interests.

## Authors' contributions

EG-V carried out the acquisition of data, analysis and interpretation of data, and drafted the manuscript. BL performed the structural analysis and interpretation of data, and drafted the manuscript. AM-A conceived the study, and participated in its design and coordination, the interpretation of data, and drafted the manuscript. All authors read and approved the final manuscript.

## Supplementary Material

Additional file 1 This excel document contains additional information about: sheet 1) the type of regulatory interactions on the network, strain, experimental evidences and references; sheet 2) the components and length of directed paths in the whole network; sheet 3) the components and length of directed paths in the regulatory sub-network; sheet 4) representative motifs in the entire network with two, three and four genes; sheet 5) components and measures of the whole network and the sub-network; sheet 6) orthologs TFs with auto-regulation identified in *E. coli*. Click here for file

Additional file 2 This is a poster version of Figure 2 to better appreciate the name of genes which are difficult to distinguish in the corresponding figure in the ms. Click here for file
