# Supplementary figures and images for: The Regulatory Network of Pseudomonas aeruginosa
Source: Microb Inform Exp. 2011 Jun 14;1:3. doi: 10.1186/2042-5783-1-3 (PMC3348663; doi:10.1186/2042-5783-1-3)

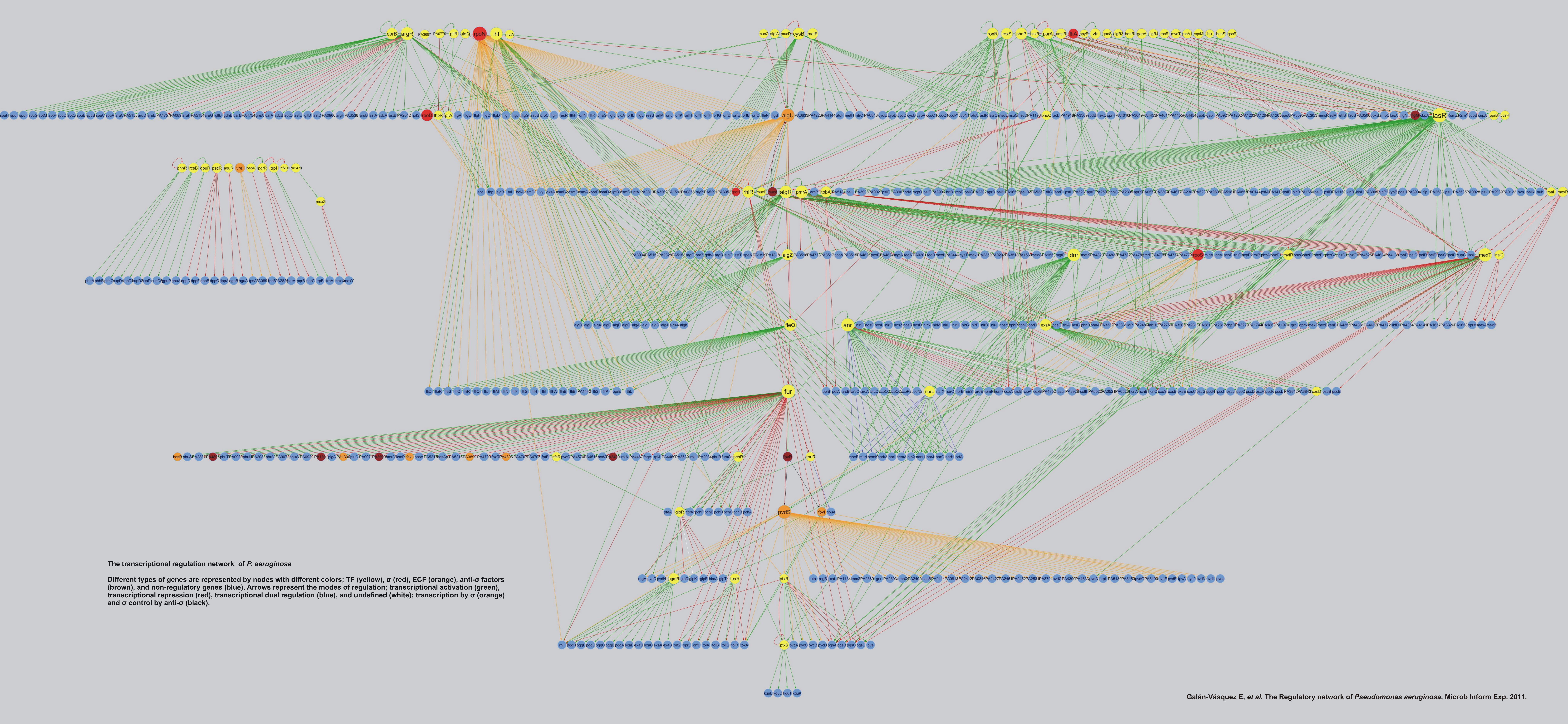

Supplement: Additional file 2 — This is a poster version of Figure 2 to better appreciate the name of genes which are difficult to distinguish in the corresponding figure in the ms. [file 2042-5783-1-3-S2.JPEG]
